# Travel distance and potential disparities in palliative radiotherapy access for cancer patients in Victoria, Australia

**DOI:** 10.1007/s00066-025-02418-8

**Published:** 2025-08-22

**Authors:** Maike Trommer, Piers Gillett, Fanny Franchini, Karen Trapani, Colin Hornby, Skye Abraham, Dishan Herath, Karla Gough, Keith Donohoe, Phillip Tran, Farshad Foroudi, Maarten IJzerman, Richard Khor

**Affiliations:** 1 Austin Health, Department of Radiation Oncology, Olivia Newton-John Cancer Wellness & Research Centre, Melbourne, Australia; 2 Faculty of Medicine and University Hospital Bonn, Department of Radiation Oncology, University of Bonn, Bonn, Germany; 3 Center for Integrated Oncology (CIO), University Hospitals Aachen Bonn Cologne Dusseldorf, Teutoburger Str. 5, 50678 Cologne, Germany; 4 Cancer Health Services Research (CHSR), University of Melbourne, Melbourne, Australia; 5 Department of Radiation Oncology, Peter MacCallum Cancer Centre, Melbourne, Australia; 6 Western Health, Cancer Services, Melbourne, Australia; 7 Department of Health Services Research, Peter MacCallum Cancer Centre, Victoria 3000, Melbourne, Australia; 8 Sir Peter MacCallum Department of Oncology, The University of Melbourne, Victoria 3010, Melbourne, Australia; 9 Department of Nursing, The University of Melbourne, Victoria 3010, Melbourne, Australia; 10 Royal Melbourne Hospital, Victorian Comprehensive Cancer Centre Alliance, Melbourne, Australia; 11 Erasmus School of Health Policy & Management, Rotterdam, The Netherlands; 12 Department of Medical Imaging and Radiation Sciences, Monash University, Melbourne, Australia; 13 School of Molecular Sciences, La Trobe University, Melbourne, Australia

**Keywords:** Palliative radiotherapy, Disparities in radiotherapy access, Travel distance, Socioeconomic status, Cancer healthcare equity

## Abstract

**Background:**

Palliative radiotherapy (PRT) is crucial for improving quality of life in patients with advanced-staged cancer. This large data analysis investigates the travel distances and potential disparities in PRT access especially focusing on the burden of excess travel for palliative cancer patients in Victoria, Australia.

**Methods:**

Using a state-wide linked dataset from the PRedicting the health economic IMPact of new and current Cancer Treatments (PRIMCAT) research program, we analysed the estimated road travel distance (ERTD) and potential excess travel distance (PETD) as well as received radiotherapy fractions for 29,807 PRT patients being treated from 2010–2019. We examined disparities by socioeconomic status (SEIFA) and remoteness (RA) of the residential area of PRT patients, and receiving treatment at a public or private centre.

**Results:**

The average one-way ERTD for all PRT patients was 43 km, with variations based on SEIFA and RA. Patients in the lowest SEIFA quintile and those living in outer regional areas had the longest ERTD. Approximately 50% did not receive treatment at the closest facility, with a mean PETD of 27.9 km for private and 24.3 km for public facility patients. Fractionation patterns showed no significant reduction in the number of fractions with increased travel distance. Patients at private facilities received more fractions on average (8.49) compared to those at public facilities (5.91).

**Conclusion:**

This study highlights potential disparities in PRT access in Victoria, with patients living in socioeconomically disadvantaged and remote regions facing longer travel distances and excess travel. These findings underscore the need for strategic referral practices and further research to optimise equitable access to PRT.

**Supplementary Information:**

The online version of this article (10.1007/s00066-025-02418-8) contains supplementary material, which is available to authorized users.

## Background

Palliative radiotherapy (PRT) plays a major role in cancer care to improve quality of life and sometimes survival outcomes in advanced cancer scenarios [1]. Palliative radiotherapy is used in patients with incurable cancer for a broad range of indications to reduce symptoms such as pain, obstruction or bleeding, with minimal toxic effects. For example, in patients with painful bone metastases, PRT reduces pain in 60% of patients and achieves complete pain relief in 25% of cases [2]. The total radiotherapy application time should be as short as possible, with shorter treatments having been shown in selected metastatic sites such as bone to elicit similar symptom control to longer courses [3, 4].

Typically, PRT regimens are tailored to the patient’s needs, focusing on maximising comfort and symptom relief. Treatment courses vary, ranging from a single dose to multiple fractions, depending on factors such as the patient’s symptoms to treat, overall health status and the type of cancer. Common fractionation schemes are 20 or 25 Gy in five fractions or 30 Gy in ten fractions or 8 Gy in a single dose [5–9].

Research has revealed both clinical and non-clinical barriers to receiving PRT, including geographical factors: a variety of studies have highlighted that an increasing distance to healthcare facilities is associated with poorer oncological outcomes and quality of life for patients. Similarly, there seems to be an inverse association between living further from a radiotherapy centre and being likely to receive PRT in Global North countries such as Canada, USA, UK, Norway and France [10, 11]. This analysis is focused on travel distances of PRT patients in Victoria, which has an area similar to that of the UK and a population which is comparable to those of Denmark and Austria [12].

This study aims to investigate the factors influencing access to PRT in Victoria, Australia. The analysis was driven by concerns about potential disparities in access, particularly among socioeconomically disadvantaged and geographically remote populations. In this study, the estimated road travel distance (ERTD) of PRT patients was calculated using a state-wide linked dataset over a ten-year period. We aimed to identify and quantify potential excess travel distances (PETD) if patients could have been treated at a closer centre. We also investigated potential disparities in subgroups regarding living in socioeconomically more or less advantaged areas (SEIFA) and remote areas (RA). Understanding these factors is critical for identifying barriers to equitable care and informing strategies to reduce the travel burden for vulnerable populations.

## Materials and methods

The analysis was confined to descriptive outcomes, and the extensive volume of cases included precluded the calculation of statistically significant differences.

### Data sources and analysis

This study utilised a state-wide linked dataset in Victoria, Australia, underpinning the PRedicting the health economic IMPact of new and current CAncer Treatments (PRIMCAT) research program. PRIMCAT is a multi-institution research initiative led by the Cancer Health Services Research Unit at the University of Melbourne, employing a data-driven modelling approach to analyse and predict cancer treatment utilisation in Australia. The dataset includes individuals aged 18 years and older at diagnosis registered in the Victorian Cancer Registry with diagnosis between 01 January 2010 and 31 December 2019. Patients were treated at both public or private facilities, and breast, prostate, lung and colorectal cancer patients were included. Treatment data were derived from the Victorian Radiotherapy Minimum Dataset [13].

All analyses were conducted using RStudio (v4.3.0; R Core Team 2023) [14].

### Estimated road travel distance

A table of postcode-to-postcode centroid travel distances and times was generated using the Google Distance Matrix API and was accessed through the gmapsdistance package using R statistical software [15, 16]. For all patients accessing PRT, each residential postcode was queried against the postcodes of the 54 radiotherapy centres which were open during the study period. Each query assumed travel distance by car; toll roads were not excluded, and the respective origin and destination was the postcode latitude and longitude coordinates of the postcode centroid. The lookup table was then used patient by patient to identify the estimated travel distance for them to attend their radiotherapy.

The ERTD was calculated as one-way travel per treatment for the four most common cancer types (breast, prostate, lung, colorectal), sex (male, female), public/private treating radiotherapy centre, socioeconomic status (SES) and remoteness of living (RL). The Victorian radiotherapy centres where the patients were treated are all well equipped to treat the common tumour types included in this analysis, with well-established treatment paradigms based on national guidelines [17].

### Socioeconomic indexes for areas and remoteness areas

The Australian Statistical Geography Standard (ASGS) classification of Remoteness Areas (RA) in Australia was used for RL [18]. The ASGS is a set of geographic data for analysis. It comes from the Australian Bureau of Statistics (ABS) and other organisations [15]. The scale is determined by the distance from large cities and access to services, and includes five categories (1 major cities/metropolitan, 2 inner regional, 3 outer regional, 4 remote and 5 very remote Australia). Victoria contains mainly people living within regions 1–4. However, the number of patients having treatment while residing in regions 4 and 5 (remote and very remote Australia) was small, and, subsequently, the results had to be censored from analysis due to risk of reidentification. In Tables 1 and 2, the numbers of patients residing in regions 1–3 are shown.

**Table 1:** Patient characteristics

Characteristic	All patients (*n*)	Palliative patients (*n*)	% of PRT patients
*n*	81,197	29,807	–
*Gender*			
Male	35,803	16,292	54.7
Female	45,394	13,515	45.3
*Age (years)*			
Mean	65.94	68.3	–
Median	67.09	69.1	–
Min	–	22.25	–
Max	–	105.7	–
25th percentile	–	57.7	–
75th percentile	–	74.8	–
*Treatment centre*			
Public	–	20,081	68.4
Private	–	9294	31.6
N/A	–	432	–
*Tumour type*			
Breast	–	5856	19.6
Colorectal	–	3667	12.3
Lung	–	15,339	51.5
Prostate	–	5975	20.0
*RA*			
Metropolitan	–	21,140	70.1
Inner regional	–	6754	22.7
Outer regional	–	1584	5.3
N/A	–	316	1.1
*SEIFA*			
1	–	7336	24.6
2	–	6032	20.2
3	–	5616	18.8
4	–	5430	18.2
5	–	5116	17.2
N/A	–	277	0.9

*PRT* palliative radiotherapy, *RA* remoteness areas, *SEIFA* socioeconomic indexes for areas, *1* = most disadvantaged, *N/A* not available

**Table 2:** Average number of fractions of a) all palliative patients, b) patients treated at a private centre not traveling to their closest facility and c) patients treated at a public centre not traveling to their closest facility

Average number of fractions	a) Group: all patients			b) Group: excess private			c) Group: excess public		
	*n*	Average fractions	Sd fractions	*n*	Average fractions	Sd fractions	*n*	Average fractions	Sd fractions
Total	29,807	6.93	5.67	4500	8.49	6.58	10,177	5.91	5.03
*Gender*									
Male	16,292	6.93	5.52	2666	8.70	6.26	5354	6.06	5.18
Female	13,515	6.93	5.84	1834	8.17	7.02	4823	5.74	4.86
*Tumour type*									
Breast	6277	7.47	6.35	917	8.56	6.75	2122	5.98	5.16
Colorectal	3836	7.93	6.24	622	9.04	6.70	1190	7.02	5.86
Lung	15,527	6.32	5.00	1715	7.25	6.13	5970	5.62	4.66
Prostate	5981	7.38	6.03	1544	9.47	6.60	1453	6.28	5.48
*RA*									
Metropolitan	21,140	6.95	5.68	3575	8.42	6.56	7841	5.82	4.95
Inner regional	6754	6.92	5.61	608	8.44	6.32	1673	6.37	5.35
Outer regional	1584	6.68	5.75	274	9.37	6.93	521	6.10	5.40
N/A	316	6.70	5.86	39	9.21	9.52	135	5.08	3.97
*SEIFA*									
1	7336	6.69	5.40	664	8.69	6.48	2252	5.79	4.93
2	6032	6.92	5.58	757	7.87	5.83	2080	6.03	5.01
3	5616	7.01	5.69	827	8.49	6.37	1983	5.92	4.95
4	5430	7.04	5.93	1000	8.56	7.48	1971	5.99	5.09
5	5116	7.07	5.81	1208	8.67	6.28	1783	5.87	5.26
N/A	277	7.02	6.26	44	9.27	9.52	108	5.30	4.11

*RA* remoteness areas, *SEIFA* socioeconomic indexes for areas, *1* = most disadvantaged, *N/A* not available, *Sd *standard deviation

We analysed travel distances considering the socioeconomic indexes for areas (SEIFA) for SES [19]. SEIFA combines census data such as income, education, employment, occupation, housing and family structure to summarise the socioeconomic characteristics of an area. Patients were allocated to five SEIFA quintiles with ‘1’ being the most disadvantaged 20% and ‘5’ the most advantaged 20% of the population.

### Potential excess travel distance

This study scrutinised both the ERTD and PETD for patients undergoing PRT. We defined PETD as the difference in distance between the ERTD of the patient’s actual RT centre and the ERTD of the closest available centre that was open at the time of treatment initiation. We performed subgroup analyses for patients receiving care at a public facility not travelling to their closest public facility as well as for patients receiving care at a private facility not travelling to their closest facility. In the Australian context, the latter reflects the fact that patients who can afford to pay out-of-pocket costs for treatment at a private centre will also have access to public facilities.

### Fractionation

To evaluate the treatment patterns across varying distances, the number of fractions and the relative proportion of fractions received according to ERTD was analysed in 25-km intervals. The fractions were categorised into common fractionation schemes, i.e., single dose, 2–5 fractions, 6–10 fractions and more than 10 fractions, to ascertain the distribution of treatment intensity relative to the distance from the radiotherapy centre [5–9].

## Results

### Patient characteristics

Out of 201,804 unique patients in the linked dataset, there were 81,197 unique courses of radiotherapy available for analysis; 29,807 (36.7%) of these were of palliative intent and included in the analysis. The analysis of PRT comprised 13,515 (45.3%) female and 16,292 (54.7%) male patients; 20,081 (68.4%) patients received treatment at a public facility, while 9294 (31.6%) received treatment at a private facility (Table 1).

Some patients in the dataset had more than one primary tumour type.

### Estimated road travel distance

All PRT patients had an average ERTD of 43 km one way, with a median of 17.9 km, which resulted in an average travel time of 38.3 min (median 22.4). Regarding their residential address, metropolitan patients had an average ERTD of 18.5 km (median 13.4), inner regional patients of 72.6 km (median 63.5) and outer regional patients of 241 km (median 214.8). Patients within SEIFA quintile 1 had an average ERTD of 54.9 km (median 21.5) and SEIFA quintile 5 of 19.5 km (median 10.9). Detailed ERTD data of all palliative patients are summarised in Supplementary Table 1.

### Potential excess travel

**Fig. 1:**
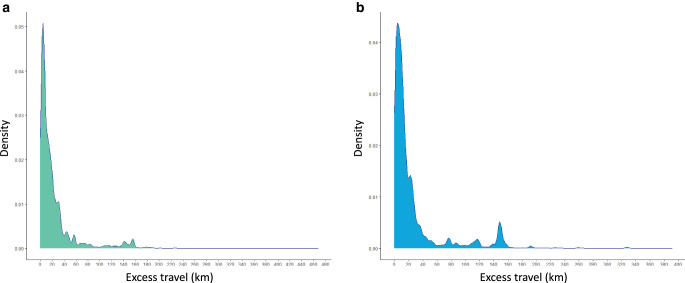
**a** Potential excess travel distances (PETD; km) of palliative patients attending a public facility (*n* = 10,177); **b** PETD (km) of palliative patients attending a private facility (*n* = 4500)

Overall, 15,125 (50.7%) patients had PETD and did not receive treatment at their closest facility. Of all patients receiving care at a private facility, 4500 (48.4%) had PETD with a mean of 27.9 km (median 11.1). This resulted in a calculated average excess travel time of 22 min (median 12.5). Patients living in metropolitan areas had a mean PETD of 12.5 km, inner regional patients of 70.2 km and outer regional patients of 131 km. The mean PETD within the SEIFA quintile 1 was 44.2 km and in the SEIFA quintile 5 it was 12.7 km. Detailed PETD data of patients attending a private facility are summarised in Supplementary Table 2 and Fig. 1a.

In total, 10,177 (50.7%) patients attending a public facility did not travel to their closest public facility. The mean PETD of those patients was 24.3 km (median 11.6), with a corresponding mean excess travel time of 18.6 min (median 11.6).

Patients receiving care at a public facility and living in metropolitan areas had an average PETD of 13.6 km, inner regional patients of 47.4 km and outer regional patients of 109 km. Patients within SEIFA quintile 1 had a mean PETD of 31.5 km, in SEIFA quintile 2 of 30.2 km, in SEIFA quintile 3 of 25.8 km, in SEIFA Quintile 4 of 18.2 km and in SEIFA quintile 5 of 13.2 km.

Supplementary Table 2 and Fig. 1b show the PETD for attending a public facility and travelling to a public centre that was not the closest.

### Fractionation

Fractionation in relation to distance showed that patients travelling less than 100 km (*n* = 26,396) had an average of 7 fractions, whereas patients traveling 100 km or more (*n* = 3411) had an average of 6.8 fractions. In general, patients receiving treatment at a private facility received more fractions on average (8.49) than those receiving treatment at a public centre (5.91).

Regarding the area of living, patients attending a private facility and living in metropolitan areas with a potential travel excess were treated with an average of 8.4 fractions, those from inner regional areas with 8.4 fractions and those from outer regional with 9.4 fractions.

Patients attending a public facility living in metropolitan areas with a potential travel excess had 5.8 fractions on average, those from inner regional 6.4 fractions and those from outer regional 6.1 fractions.

No differences in fractionation by SEIFA were observed (Table 2).

**Fig. 2:**
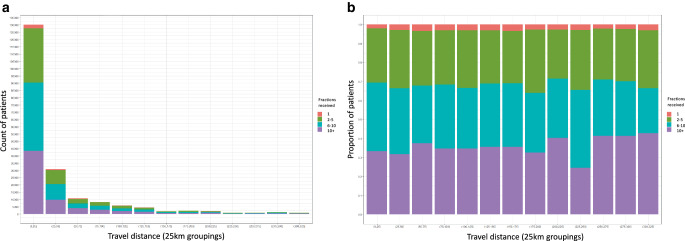
Number (**a**) and relative proportion (**b**) of fractions received by patients per estimated road travel distance. Fractions were grouped into single fraction (red), 2–5 fractions (green), 6–10 fractions (turquoise) and more than 10 fractions (purple)

Grouping all patients by ERTD in 25-km intervals shows no change in the median number of fractions with increasing ERTD (5.00). Figure 2 shows the number of fractions and relative proportion of fractions received by all patients per ERTD in 25-km intervals.

## Discussion

To the best of our knowledge, this is the largest analysis of travel distances for Australian cancer patients receiving PRT. The aim was to identify potential disparities in access to PRT. While most studies regarding travel distances and RT are focused on the likelihood of a patient receiving RT at all, this study focuses primarily on the burden of excess travel for the vulnerable patient population being treated with PRT [20–23]. Specifically, we set out to estimate likely travel distances and quantify PETD and fractionation considering different subgroups. There is existing evidence for an association between both increased travel distance to healthcare facilities and socioeconomic disadvantage and poorer health outcomes [10]. Victoria is the smallest of Australia’s mainland states in area and covers about 237,659 km^2^. Victoria’s area is similar to that of the UK and has a population of approximately 6.7 million, which is comparable to Denmark (5.9 million) and Austria (8.5 million), with Melbourne comprising 5.2 million inhabitants. However, geographic and population density comparisons need to be viewed in the context of different healthcare systems, which would be beyond the scope of this article to present in detail.

In our cohort, patients had an average ERTD of 43 km (median 18), corresponding to a calculated 38 min (median 22) travel time. Differences in ERTD in this study were especially present in female patients and breast cancer patients who had the shortest travel distance. Prostate cancer patients had the longest ERTD. There are various possible reasons for the differences in ERTD. The indication for PRT treatment varies with tumour type. For instance, PRT to primary prostate cancer may have a survival advantage [24]. There is also an OS benefit in treatment of oligometastatic lung cancer [25].

It is important to realise that ERTD only estimates one-way travel. The maximum theoretical weekly travel distance if patients travel each day for treatment could be as high as a two-way commute multiplied by the number of fractions (e.g. using the median ERTD of 16 km and median fractions of 7, the course-based ERTD would be 224 km). The impact on patient quality of life is not captured fully using ERTD alone. In Victoria, approximately 29,500 radiotherapy fractions were for palliative purposes in the 2018/2019 period [26].

### Excess travel for PRT

The data reveal a notable trend, where more than 50% of the analysed patients underwent PRT at a centre that was not the closest centre to their residential address. This may be explained by several factors. Referral patterns of healthcare professionals and patient preferences based on personal experience could influence this decision-making process. Healthcare professional referral patterns were recently discussed in a cross-sectional survey amongst palliative care physicians by Vargas et al., who reported one of the top four perceived barriers to referral for PRT to be the distance to the radiotherapy centre [27]. Physicians from more remote centres were more likely to be concerned about the distance to radiotherapy facilities and the lack of a radiation oncologist available for discussing cases. Even though palliative care physicians have a pivotal role in referring patients for PRT, they also pointed out that there might be a lack of referral because of insufficient knowledge about it [19]. This was also confirmed by Leiss et al., who published a survey in 2025 highlighting a paradox whereby general practitioners show high engagement in palliative care but limited involvement in RT management due to communication gaps and professional development needs [28]. In our study, we could not analyse the proportion of patients who were actually referred to PRT or treated with other palliative therapies. This could be subject of more detailed utilisation studies in the future. Nevertheless, referral patterns are a very important issue to discuss in this context and should be addressed in future studies.

Additional factors that could not be explored within the limitations of the dataset were the waiting list or appointment availability, participation in clinical trials, support services, and logistical considerations such as transportation and lodging options near more distant facilities. Subgroup analyses of patients receiving treatment at a private or public facility who did not attend their closest treatment centre were performed. Nearly half of the private patients (48.4%) and over half of the public patients (50.7%) incurred potentially excess travel. Notably, the calculated corresponding excess travel time averaged around 22 min for private patients and 18.6 min for public patients.

The high excess travel but also the ERTD is especially remarkable considering the number of 54 existing radiooncology centres in Victoria, whereas Zurl et al. reported in 2018 that for a population of 8.51 million in Austria, 43 MV units in 14 centres were at disposal [12]. Unfortunately, they did not report travel distances, but their survey revealed major regional differences in radiooncological capacity regarding, e.g. waiting times.

### Socioeconomic status and remoteness

Regarding RL—defined using RA and SES—defined using SEIFA, our study revealed differences in patients, whether treated at a private or a public facility. The lower the SEIFA quintile (greater disadvantage), the longer the observed average ERTD and PETD for patients. The more remote their residential region, the longer the ERTD, which was expected, but also higher PETD was observed. Patients in outer regional areas and with greater disadvantage had the longest ERTD and also the longest PETD on average. Thompson et al. [20] asserted that distance still plays an important role in influencing the uptake of radiation therapy, but it is not the only issue. They stressed that the complex nuances between socioeconomic, cultural and health system factors that influence patients’ decision-making bear further consideration. Also carer support, the duration of displacement from home and the financial impact of the required care could be practical factors influencing a patient’s selection of treatment centre [29].

Our findings align with evidence that patients from rural areas and socially disadvantaged backgrounds generally face the longest travel times, whether they travel by public transport or by car, as evidenced by Han et al., who analysed prostate cancer treatment in the UK [30]. Similarly, an analysis of 52,317 women with breast cancer in the US revealed that patients living in rural areas travelled on average nearly three times further than those from urban areas, and their nearest facility was more than four times further away. In contrast to the UK study, the US analysis found that lower household income was associated with shorter actual travel distances, most likely related to higher income being associated with access to distant private facilities [31]. However, this comparison has to be made with caution because of the different healthcare systems: radiotherapy treatment in the US could be affected by the fact that health insurance is limited to certain centres.

In our study, we did not only evaluate the ERTD for PRT indications but also calculated the potential excess travel. SEIFA allocation was inversely related to excess travel, with patients residing in more socioeconomically disadvantaged areas enduring longer excess travels. This is consistent with the fact that patients who can afford out-of-pocket costs for radiotherapy may potentially have treatment at public or private facilities, which logically may decrease their distance to their closest centre due to higher centre geometrical “density.” The inverse relationship between SEIFA and PETD highlights a socioeconomic gradient in healthcare access, suggesting that those in lower socioeconomic areas face greater barriers to receiving care. This disparity may exacerbate existing health inequities. The reasons why a remarkable proportion of patients living in lower socioeconomic areas (almost 50% of PRT patients live in SEIFA 1 and 2) and more remotely do not receive PRT at their closest centre needs further investigation.

### Radiotherapy fractionation

Patients in this study received 6.9 fractions on average. Giving fewer RT fractions in the palliative setting is in line with the common practice to minimise the treatment-related burden for patients with a limited life expectancy. Notably, there was no meaningful difference in the number of fractions for patients who lived in regional areas. As distance to the treatment centre increases, the number of fractions does not decrease. Given the logistical challenges and potential stress associated with travel, it would be anticipated that palliative patients coming from afar would be more likely to receive a reduced number of fractions, possibly just a single dose [2, 32–34]. Nor did SEIFA have an impact on fractionation in our analysis. These observations suggest a potential area for further investigation into the factors influencing radiotherapy fractionation and the consistency of applying guidelines across different geographic locations. A study by Ong et al. also discussed this topic, mainly focusing on the use of single-fraction radiotherapy (SFRT) for bone metastases in Australia [35]. They found that SFRT, a well-established, efficient and less burdensome approach for palliative radiotherapy (especially for bone metastases), was underutilised in Victoria, with only 17% of patients receiving this treatment. This suggests a potential gap between clinical guidelines and real-world practice.

Study findings underscore the complexity of radiotherapy access in the palliative care context. The significant proportion of patients undertaking excess travel, as well as the distance patients from more remote and socioeconomically disadvantaged backgrounds may travel, emphasises the need for more strategic referrals to radiotherapy centres to reduce the travel burden in these vulnerable populations. In-depth investigation is required to gain a comprehensive understanding of the distinct travel patterns exhibited by PRT patients. Further research is needed to unravel the complexities of patients’ travel patterns. Given that calculating ERTD is feasible, it may be possible to assign a cost to travel (and excess travel) to furnish ongoing discussions about value-based healthcare. It is crucial that patients can access radiotherapy affordably, quickly and conveniently. Travelling greater distances incurs higher expenses, which are an often-overlooked aspect of referral and treatment and can cause financial burden. Financial toxicity is a highly relevant topic for cancer patients, as indexed in numerous studies across the globe, and which is even associated with worse oncological outcomes [36–38]. Understanding the intricate reasons behind travel patterns would necessitate further exploration to unveil the nuanced decision-making processes of palliative cancer patients and their healthcare teams.

The study highlights several key recommendations. Educating healthcare professionals about local RT options is essential, as over 50% of patients did not receive PRT at the closest facility. This could reduce unnecessary travel burdens. Addressing healthcare inequalities is also crucial, as patients from socioeconomically disadvantaged or remote areas face longer travel distances and excess travel. Our data imply that because increasing social disadvantage is associated closely with RL, future research and policy focussing on socioeconomic determinants of RT is likely to be incomplete without the inclusion of regionality indices. Policymakers should prioritise equitable access by improving regional services, funding travel subsidies and expanding specialised care to underserved areas.

To improve access, clear patient education on treatment options, better data on transport availability and targeted resource allocation are needed. These measures would reduce travel burdens and healthcare inequities while ensuring convenient and affordable radiotherapy access.

### Limitations

The study’s reliance on postcode-centroid-to-postcode-centroid calculations may underestimate actual travel distances. This potential underestimation, combined with assumptions such as the > 100-km threshold, could impact our findings. Due to the relatively small number of patients in the > 150-km travel distance categories, results should be interpreted with caution; however, we chose to present these separately to preserve granularity and highlight the considerable travel distances undertaken by some patients in Fig. 2a. Our analysis did not consider how travel distance impacts patient outcomes, transportation choices or accommodation decisions. For instance, while some patients might have opted to travel further to stay with family near their treatment facilities, our calculations assumed that they travelled from their residential addresses. We did not explore the effects of PETD on quality of life or related costs. Additionally, our analysis did not account for the specific times at which PRT was administered, meaning that the travel times estimated might not reflect peak or off-peak travel conditions.

## Conclusion

This analysis of the travel distances of 29,807 palliative cancer patients receiving PRT in Victoria, Australia, reveals meaningful insights into travel differences and associated disparities. More than 50% received PRT at a centre that was not their closest and travelled up to 468 km maximum PETD. The ERTD increased with lower SES and RL of the residential area of the patients. The PETD commensurately increased with lower SES and RL. Patients treated at a private centre received more fractions on average than patients at public centres, while fractionation stayed the same with increasing distance and SES. Our findings underscore the complexity of healthcare access in the palliative care context. The consistency in PRT fractionation despite increased distance highlights a potential area for optimisation and further investigation. Addressing these challenges requires a multifaceted strategy that considers geographic, economic and clinical factors to ensure equitable access to PRT.

## Supplementary Information

Supplementary Table 1: Estimated road travel distance (km) of all palliative patients

Supplementary Table 2: PETD (km) of palliative patients receiving care at a private or public facility and travelling to a facility that was not their closest
