# Supplementary material for: Travel distance and potential disparities in palliative radiotherapy access for cancer patients in Victoria, Australia
Source: Strahlenther Onkol. 2025 Aug 22;201(12):1296–305. doi: 10.1007/s00066-025-02418-8 (PMC12701006; doi:10.1007/s00066-025-02418-8)
Supplement: Supplementary file 1 — Supplementary Table 1: Estimated road travel distance (km) of all palliative patients [file 66_2025_2418_MOESM1_ESM.docx]

**Supplementary Table 1:** Estimated road travel distance (km) of all palliative patients.

| **Distance (km)** | **N** | **mean** | **median** | **min** | **max** | **IQR** | **q25** | **q75** | **q95** |
| --- | --- | --- | --- | --- | --- | --- | --- | --- | --- |
|  |  |  |  |  |  |  |  |  |  |
| **One-way** |  |  |  |  |  |  |  |  |  |
| Total | 29,807 | 43.06 | 17.88 | 0 | 630.22 | 33.52 | 8.42 | 41.94 | 179.57 |
|  |  |  |  |  |  |  |  |  |  |
| **Gender** |  |  |  |  |  |  |  |  |  |
| male | 16,292 | 44.90 | 18.09 | 0 | 630.22 | 35.32 | 8.42 | 43.74 | 185.47 |
| female | 13,515 | 40.83 | 17.60 | 0 | 588.96 | 31.49 | 8.42 | 39.91 | 165.57 |
|  |  |  |  |  |  |  |  |  |  |
| **Tumour stream** |  |  |  |  |  |  |  |  |  |
| breast | 6,277 | 38.91 | 16.52 | 0 | 588.43 | 28.70 | 8.23 | 36.93 | 152.99 |
| colorectal | 3,836 | 44.76 | 17.60 | 0 | 588.96 | 33.04 | 8.41 | 41.45 | 198.41 |
| lung | 15,527 | 42.73 | 18.09 | 0 | 630.22 | 33.72 | 8.42 | 42.14 | 176.56 |
| prostate | 5,981 | 46.60 | 17.86 | 0 | 588.96 | 38.58 | 8.15 | 46.73 | 188.12 |
|  |  |  |  |  |  |  |  |  |  |
| **RA** |  |  |  |  |  |  |  |  |  |
| metropolitan | 21,140 | 18.49 | 13.40 | 0 | 580.27 | 15.78 | 7.33 | 23.11 | 47.63 |
| inner regional | 6,754 | 72.57 | 63.55 | 0 | 630.22 | 84.64 | 20.56 | 105.20 | 193.48 |
| outer regional | 1,584 | 240.97 | 214.79 | 0 | 588.96 | 178.72 | 130.52 | 309.24 | 550.66 |
| N/A | 316 | 50.75 | 18.35 | 0 | 551.19 | 44.50 | 8.42 | 52.92 | 211.29 |
|  |  |  |  |  |  |  |  |  |  |
| **SEIFA quintile** |  |  |  |  |  |  |  |  |  |
| 1 | 7,336 | 54.87 | 21.49 | 0 | 588.96 | 54.92 | 8.63 | 63.55 | 218.95 |
| 2 | 6,032 | 55.54 | 23.47 | 0 | 588.96 | 56.05 | 10.19 | 66.24 | 206.80 |
| 3 | 5,616 | 44.98 | 19.70 | 0 | 562.18 | 36.47 | 8.72 | 45.19 | 185.47 |
| 4 | 5,430 | 32.94 | 15.54 | 0 | 630.22 | 22.13 | 8.32 | 30.45 | 130.52 |
| 5 | 5,116 | 19.54 | 10.85 | 0 | 551.19 | 13.64 | 6.54 | 20.18 | 64.41 |
| N/A | 277 | 52.03 | 20.00 | 0 | 551.19 | 43.82 | 8.42 | 52.24 | 223.25 |

RA = Remoteness Areas; SEIFA = Socio-Economic Indexes for Areas, 1 = most disadvantaged; N/A = not available.
