# Supplementary material for: Travel distance and potential disparities in palliative radiotherapy access for cancer patients in Victoria, Australia
Source: Strahlenther Onkol. 2025 Aug 22;201(12):1296–305. doi: 10.1007/s00066-025-02418-8 (PMC12701006; doi:10.1007/s00066-025-02418-8)
Supplement: Supplementary file 2 — Supplementary Table 2: PETD (km) of palliative patients receiving care at a private or public facility and travelling to a facility that was not their closest [file 66_2025_2418_MOESM2_ESM.docx]

**Supplementary Table 2:** PETD (km) of palliative patients receiving care at a private or public facility and travelling to a facility that was not their closest.

|  | **N** | **mean** | **median** | **min** | **max** | **IQR** | **q25** | **q75** | **q95** |
| --- | --- | --- | --- | --- | --- | --- | --- | --- | --- |
| **Distance Excess (km) Private** | | | | | | | | | |
| **One-way** |  |  |  |  |  |  |  |  |  |
| Total | 4,500 | 27.93 | 11.10 | 0.00 | 391.66 | 19.42 | 4.99 | 24.41 | 147.60 |
| **Gender** |  |  |  |  |  |  |  |  |  |
| male | 2,666 | 29.17 | 11.43 | 0.00 | 332.43 | 20.75 | 5.15 | 25.90 | 148.12 |
| female | 1,834 | 26.14 | 10.46 | 0.00 | 391.66 | 18.30 | 4.83 | 23.13 | 138.92 |
| **Tumour stream** |  |  |  |  |  |  |  |  |  |
| breast | 917 | 25.96 | 9.90 | 0.00 | 328.08 | 17.67 | 4.54 | 22.21 | 136.21 |
| colorectal | 622 | 30.43 | 10.59 | 0.00 | 366.05 | 22.19 | 4.53 | 26.72 | 147.60 |
| lung | 1,715 | 26.65 | 11.45 | 0.00 | 391.66 | 18.96 | 5.18 | 24.14 | 140.63 |
| prostate | 1,544 | 29.04 | 11.09 | 0.00 | 332.43 | 20.54 | 5.14 | 25.68 | 148.87 |
| **RA** |  |  |  |  |  |  |  |  |  |
| metropolitan | 3,575 | 12.54 | 8.75 | 0.00 | 218.87 | 11.73 | 3.91 | 15.64 | 35.75 |
| inner regional | 608 | 70.22 | 50.47 | 0.60 | 391.66 | 85.59 | 23.89 | 109.48 | 178.06 |
| outer regional | 274 | 130.98 | 147.60 | 1.21 | 327.32 | 30.56 | 119.95 | 150.51 | 192.05 |
| N/A | 39 | 46.75 | 9.20 | 0.23 | 266.00 | 34.77 | 5.82 | 40.59 | 182.71 |
| **SEIFA quintile** |  |  |  |  |  |  |  |  |  |
| 1 | 664 | 44.21 | 14.38 | 0.24 | 332.43 | 49.72 | 6.05 | 55.77 | 150.51 |
| 2 | 757 | 38.52 | 15.63 | 0.00 | 391.66 | 34.83 | 7.16 | 41.99 | 148.12 |
| 3 | 827 | 31.81 | 12.20 | 0.00 | 328.08 | 21.53 | 6.02 | 27.55 | 147.60 |
| 4 | 1,000 | 23.67 | 11.62 | 0.06 | 327.32 | 16.93 | 6.42 | 23.35 | 117.91 |
| 5 | 1,208 | 12.71 | 7.38 | 0.00 | 189.82 | 11.43 | 2.90 | 14.33 | 40.13 |
| N/A | 44 | 41.96 | 9.20 | 0.23 | 266.00 | 31.24 | 5.82 | 37.06 | 171.04 |
| **Distance Excess (km) Public** | | | | | | | | | |
| **One-way** |  |  |  |  |  |  |  |  |  |
| Total | 10,177 | 24.29 | 11.63 | 0.03 | 467.89 | 21.41 | 4.27 | 25.67 | 118.34 |
| **Gender** |  |  |  |  |  |  |  |  |  |
| Male | 5,354 | 24.41 | 11.62 | 0.04 | 467.89 | 21.17 | 4.27 | 25.44 | 122.31 |
| Female | 4,823 | 24.17 | 11.63 | 0.03 | 335.84 | 21.41 | 4.27 | 25.68 | 116.04 |
| **Tumour stream** |  |  |  |  |  |  |  |  |  |
| Breast | 2,122 | 23.10 | 10.95 | 0.05 | 296.12 | 19.91 | 4.27 | 24.18 | 116.10 |
| Colorectal | 1,190 | 24.06 | 11.05 | 0.04 | 364.28 | 19.58 | 4.68 | 24.26 | 109.46 |
| Lung | 5,970 | 24.65 | 11.75 | 0.03 | 467.89 | 22.15 | 4.27 | 26.42 | 120.66 |
| Prostate | 1,453 | 25.35 | 12.98 | 0.04 | 328.18 | 21.89 | 4.58 | 26.47 | 128.45 |
| **RA** |  |  |  |  |  |  |  |  |  |
| metropolitan | 7,841 | 13.65 | 8.25 | 0.03 | 364.28 | 14.63 | 3.95 | 18.58 | 40.54 |
| inner regional | 1,673 | 47.45 | 28.59 | 0.22 | 467.89 | 51.55 | 14.21 | 65.76 | 146.90 |
| outer regional | 521 | 109.01 | 140.44 | 0.24 | 335.84 | 118.73 | 36.74 | 155.47 | 189.63 |
| N/A | 135 | 27.08 | 7.82 | 0.04 | 330.64 | 15.93 | 4.27 | 20.20 | 126.95 |
| **SEIFA quintile** |  |  |  |  |  |  |  |  |  |
| 1 | 2,252 | 31.50 | 15.42 | 0.24 | 335.84 | 24.57 | 5.91 | 30.48 | 145.41 |
| 2 | 2,080 | 30.16 | 15.96 | 0.21 | 240.33 | 25.57 | 5.91 | 31.48 | 143.00 |
| 3 | 1,983 | 25.84 | 13.74 | 0.05 | 330.64 | 22.90 | 5.69 | 28.59 | 115.19 |
| 4 | 1,971 | 18.21 | 10.09 | 0.03 | 467.89 | 14.64 | 3.89 | 18.53 | 58.89 |
| 5 | 1,783 | 13.16 | 5.93 | 0.04 | 346.12 | 11.09 | 3.54 | 14.63 | 44.88 |
| N/A | 108 | 27.51 | 11.53 | 0.83 | 296.12 | 18.60 | 4.24 | 22.84 | 130.72 |

RA = Remoteness Areas; SEIFA = Socio-Economic Indexes for Areas, 1 = most disadvantaged; N/A = not available.
